# Reducing Implicit Cognitive Biases Through the Performing Arts

**DOI:** 10.3389/fpsyg.2021.614816

**Published:** 2021-05-17

**Authors:** Josué García-Arch, Cèlia Ventura-Gabarró, Pedro Lorente Adamuz, Pep Gatell Calvo, Lluís Fuentemilla

**Affiliations:** ^1^Cognition and Brain Plasticity Unit, Department of Cognition, Development and Educational Psychology, University of Barcelona, Barcelona, Spain; ^2^Faculty of Health and Life Sciences, University Pompeu Fabra, Barcelona, Spain; ^3^Fundació Èpica – La Fura dels Baus, Badalona, Spain; ^4^Institute of Neurosciences, University of Barcelona, Barcelona, Spain; ^5^Bellvitge Institute for Biomedical Research-IDIBELL, L’Hospitalet de Llobregat, Spain

**Keywords:** implicit bias, implicit association test, social cognition, malleability, attitudes, performing arts

## Abstract

The aim of the present research was to test whether involvement in a 14-days training program in the performing arts could reduce implicit biases. We asked healthy participants to complete an Implicit Association Test (IAT) to assess biased attitudes to physical illness in two separate sessions, before and after the training program. Two separate control groups matched by age, gender and educational level completed the two IAT sessions, separated by same number of days, without being involved in the training program. Results showed that participants who were involved in the training program reduced their implicit bias toward illness measured through IAT in the second session. This reduction in IAT measures was not observed in the control samples, despite the two IAT measures being matched in temporal delay with the experimental group. These findings suggest that an interventional program based on the performing arts could be effective in reducing levels of implicit biases among the general population.

## Introduction

Implicit biases include attitudes that form through experiences and operate outside an individual’s awareness. Due to implicit cognitive biases, people may often attribute certain qualities or characteristics to all members of a particular group, a phenomenon known as stereotyping or prejudice. The effects of implicit bias on behavior have sparked a lot of attention, particularly when it comes to discrimination in important areas of life including healthcare, law enforcement, housing, criminal justice, and education (FitzGerald et al., 2019). Given this, society is interested in figuring out how to minimize implicit bias in the general population, as well as among practitioners who work in these fields.

To this date, the most widely accepted indicator of implicit biases is the Implicit Association Test (IAT; Greenwald et al., 1998). In the IAT task, participants are asked to categorize words as referring to negative or positive valence together with either images or words. For example, concepts such as “black” or “white” people could be associated with “good” or “bad”. Response latency to this categorization decision is then calculated and used as a measure that quantifies the strength of implicit associations between categories for a given individual. The IAT has undergone extensive testing and has been shown to be insensitive to procedural variation (Nosek et al., 2005) and less susceptible to individual response biases than questionnaires (Steffens, 2004). The IAT has also been shown to be reliable across test-retest experimental designs (Bosson et al., 2000; Dasgupta et al., 2000; Greenwald and Farnham, 2000; Greenwald and Nosek, 2001).

Implicit measures were initially assumed to be hardly susceptible to change (Fazio et al., 1995). However, with multiple studies showing that implicit associations are vulnerable to laboratory interventions, this viewpoint has changed to one of implicit malleability (for reviews, see Blair, 2002; Gawronski and Bodenhausen, 2006; Lai et al., 2013). Intriguingly, a recent study that evaluated several interventional approaches showed that those that were more self-relevant, emotional, and vivid were also more effective in inducing a change in implicit cognitive biases (Lai et al., 2014). This is in line with previous research indicating that messages that are related to the one’s “self” are more likely to be processed and become personally relevant (e.g., Petty and Cacioppo, 1990; Blankenship and Wegener, 2008).

Motivated by the need to find ways to decrease levels of implicit biases among the general population, we here aimed to investigate whether the involvement of non-professional artists in a training program on the performing arts influenced implicit measures. Acting requires an actor to understand a character’s mental world and the experience of that character’s feelings. The willingness to take another person’s point of view is a critical element in social functioning that promotes empathy for others (Davis, 1983) and changes representations of an out-group to be more self-like (Galinsky and Moskowitz, 2000). Indeed, it has been shown that imagining an event from another person’s point of view increases the perception of sharing their own personality attributes (Davis et al., 1996).

Here, we asked non-professional artists to enroll in a training program of 14 consecutive full days, whereby they were requested to devise, prepare and implement a real show that expressed the notion of “cancer and neurodegenerative diseases.” In fact, adverse medical conditions are an important source of implicit cognitive biases in the population. Studies in patients under negative health conditions showed they feel stigmatized and reported feelings of exclusion, rejection or devaluation (Weiss et al., 2006). Stigma-induced discrimination in turn, has been associated with different negative outcomes such as more psychological distress (Berjot and Gillet, 2011), more depressive symptoms (Roberts et al., 2020) less self-esteem (Liu et al., 2020) and the reduction of perceived quality of life (Chambers et al., 2012, 2015).

In the current study, artists were immersed into a preparation stage that involved meeting groups of scientists that researched different aspects of the illness, from molecular biology to patient care. During the 14-days program, artists were exposed to information that allowed them to better understand the illness from a biological point of view, as well as to help them invoke realistic behavior from a patients’ perspective, thereby fostering self-relevancy, emotionality, and vividness in their training program. We hypothesized that this particular interventional approach would influence implicit cognitive bias to illness, a stigma that is extended among the general population and clinical practitioners (Link et al., 2004; Brohan et al., 2010). To quantify the degree to which the training program influenced implicit cognitive bias on an “illness,” artists (i.e., the experimental group) were requested to complete an IAT before and after the training program that included images of sick/healthy people and words related to good/bad attributes. Two matched control groups of healthy participants were also included in the study. The first control group (no intervention) completed the IAT sessions separated by the same interval of days. The second control group underwent an information-based intervention and completed the IAT sessions separated by the same interval of days. The data from the control studies was used to assess for the specificity of possible changes between the two IAT sessions in the experimental group.

## Materials and Methods

### Participants

A group of forty-six volunteers were invited to participate in the 2-session experiment separated into different days. Participants in the experimental group were selected by La Fundació Èpica based on their profiles, knowledge and professional experience. The profiles of the participants included amateur artists, scientists, neuroscientists, engineers and historians. Nineteen individuals accepted the invitation to participate but only sixteen (7 female) participants completed the two experimental sessions, which were the ones included in the analysis. Participants’ average age was 31.62 years (*SD* = 6.09). The sample of participants in the no-intervention condition included sixteen individuals matched one-by-one with the experimental group on age, gender and years of education. Thus, the sample of participants in the no-intervention condition comprised sixteen participants (7 female) with an average age of 31.62 years (*SD* = 6.09) too. Sixteen different participants were initially recruited to take part in the information intervention condition. However, two of them did not perform the second IAT session of the experiment and were excluded from the final sample. Thus, the sample of participants included in the information intervention condition comprised fourteen participants (7 female) with an average age of 29.4 years (*SD* = 6.78). The groups did not differ in age [*F*(1, 45) = 0.95, *p* = 0.33, η^2^ = 0.02]. The participants included in the two control conditions were recruited via posts on social media platforms, the group website, and email contact by the researchers of the Cognition and Brain Plasticity group. Participants from the three groups had similar educational levels [experimental group: *M* = 11.75 years, *SD* = 1; no-intervention control group: *M* = 11.87, *SD* = 1.54; information intervention control group: *M* = 11.87, *SD* = 1.54; The groups did not differ in years of education *F*(1, 45) = 0.04, *p* = 0.84, η^2^ < 0.01]. Participants in the experimental and control groups reported no history of psychiatric or neurological disease and were free of any psychoactive medication during the study. None of the participants in this study received monetary compensation for their participation. Participants in the control groups were informed about the importance of their commitment to the experiment and advised they should be able to finalize it once the experiment commenced. Participants invited to the information intervention condition were also motivated by the possibility to learn on neurodegenerative diseases and cancer. The researchers were in charge of contacting them reminding each of the appointed activities scheduled during the 14 days. The study protocol was approved by the ethics committee of the University of Barcelona (Institutional Review Board IRB00003099).

### The Performing Arts Interventional Condition

The experimental group enrolled in an interventional program lasting 14 consecutive full days, developed by la Fundació Èpica—La Fura dels Baus^1^ which, through the performing arts, is capable of effectively promoting self-engagement, vividness and emotion. The creative process applied in the program is based on the methodology and language developed by La Fura dels Baus over 40 years.

The process consists of dynamic theater activities which include scientific training. On the first day, different scientists explain via seminars and Q&A approach the scientific concepts, research questions and approaches behind cancer and neurodegenerative disorders. Artists were exposed to information that allowed them to better understand the illness from a biological point of view. The remaining 13 days are divided into three phases: the cohesion and the creation phases, and the show.

The cohesion phase takes place during the first 5 days with the objective of generating a homogeneous, solid, united and combined group among the participants. The first phase starts with a group of individuals as heterogeneous as possible, who do not know each other. During this phase, participants are immersed in a series of physical and theatrical exercises specially designed to tackle disinhibition, team building, cooperation and emotional arousal.

Specifically, the cohesion phase has the following objectives:

(1) Exercises for group consolidation and rapport: disinhibition, in groups, in pairs, individually; exercises of competition, confidence, concentration, imperative collaboration, strategy and improvisation.
(2) Warm-up and activation. Physical warm-up not only to avoid ailments and injuries but also to activate the tools of the creative process from the specific body and voice work. Active listening.
(3) Skill work in space and time. Spatial awareness, volume, proportions, qualities. Rhythm, speed, volume and physicality of sound.
(4) Action as an impulse of creation. Error as a creative resource. Active imagination.

Through the game or dynamics with simple rules as a group, we proceed to enter directly into the action with active listening exercises in which, in the face of sound, rhythmic or movement stimuli, the participants must achieve clear objectives amplified by physical actions and sounds.

The exercises are eminently cooperative. Each individual action has a reaction that affects the team, the partner or the group. Therefore, despite the existence of personal objectives, they never revert exclusively to oneself but also require common tasks to achieve collective challenges.

Another part of the creative process is based on the work of the group, the simultaneous work, development of the figure of the leader, group listening, active proposal and collective response. The exercises themselves contain motor activities that are self-regulated by the group itself.

A specific activity was implemented on days 3 and 5 to help the artist invoke realistic behavior from a patients’ and caregivers’ perspective, thereby fostering self-relevancy, emotionality, and vividness in their training program. The activity consisted of simulating 24 h of normal activity as a patient or as a caregiver. Half of the artists simulated the day-life activity of a patient with scapulo-humeral dystrophy and the other half of a patient with the amyotrophic lateral sclerosis disease.

By the end of the cohesion phase, Èpica introduces a narrative concept (i.e., cancer and neurodegenerative diseases) to work on during the next phase, and on which the final performance will be based.

The creation phase follows the cohesion phase. During the creation phase, participants are required to transform a narrative concept into a performing arts performance. During this phase, participants design and implement the specific set-up that will guide the final show. The process is highly creative and engaging, thereby bringing participants into a fully immersive environment with high doses of arousal. The creation phase has several stages: identification of skills/interests, transformation of ideas into actions, run-throughs, identification of technical needs and rehearsals.

The creation phase is based on the knowledge provided by experts in cancer and neurodegenerative diseases, and on the experience of the participants after living with the disease—both as patients and as caregivers—in order to transmit their experiences to the public. In addition, researchers could evaluate the way of feeling and experiencing their limitations in groups of individuals unfamiliar with these situations and compare them with the perceptions of the patients.

During this phase, the working groups were formed to develop a performing arts strategy to represent specific features of the diseases in the final show. These groups comprised 5–6 persons. Each group worked every day on a specific feature of the diseases and explained the outcome of the work at the end of the day to the rest of the artists.

Throughout the creation phase, group meetings were held with all the participants to define and structure the show based on the performing art strategy developed by each of the working groups. These meetings took place every 2 days to ensure the show represented a cohesive strategy of how to represent the diseases and fit to the timings of the final show.

The creation phase ended with the display of the training result, which consists of two or more rehearsals of the final show in front of a general audience of ∼100 people^2^.

During the program, scientists from research groups facilitated the acquisition of ideas related to the topic of the performing arts program, responding to questions and concerns about some of the concepts explained on the first day. Scientists from different institutions took part in the program: from the Bellvitge Biomedical Research Institute, the Department of Philosophy from the Autonomous University of Barcelona, the Germans Trias i Pujol Research Institute, and the HKU University of the Arts in Utrecht.

### The Information Intervention Condition

We designed a separate study to control for the possibility that changes in the IAT in the experimental group would be accounted only by having acquired scientific and medical notions about the diseases. The design aimed to resemble the periods of time in the 14-day program at which the experimental group received information about the diseases from scientists.

Given the limitations imposed by the COVID-19 pandemic situation in Spain during the course of this control study (March 2021), participants were requested to watch a set of lectures available on internet. The videos selected for this study included 2 lectures about cancer (50 and 18 min, respectively) and 3 lectures about neurodegenerative diseases (67, 11, and 4 min, respectively). The videos were watched on the first, second, seventh and thirteenth day of the program. To assess whether the participants maintained their attention while watching the videos as well as to give them a purpose for doing so, we informed them that they would be assessed by means of a questionnaire regarding their knowledge acquired after completing the program. This questionnaire consisted of 10 questions in a true/false format about the diseases explained in the videos. The average proportion of correct answers was *M* = 0.81 *SD* = 0.35 95% CI [0.63, 1.00] and above chance [*t*(13) = 3.35 *p* < 0.01].

### The No-Intervention Condition

A separate study was designed to control for the possibility that changes in IAT could be explained by the delayed re-exposure of the participant to the IAT task. In this control study, a separate sample of healthy volunteers were required to perform the IAT task two times separated by 14 days and no intervention was implemented in between.

### Materials

The IAT experiment included sixteen words in Spanish and eight images. Words representing “Good” attributes were “Beautiful, Precious, Contented, Enthusiasm, Excellent, Glorious, Pleasure, Happy” and words representing “Bad” attributes were “Horrible, Frightful, Hate, Grief, Selfishness, Poison, Disgust, Dirty.” Images were selected from web searches to depict four different people dressed up as patients from a hospital (“Sick” category) and four people dressed up as sporty individuals (“Healthy” category). Each picture category set included two female and two male individuals and, although the two sets of images were intended to reflect distinct contextual circumstances of people, both sets depicted people that expressed pleasant and smiling faces.

### Procedure

The IAT consisted of 7 blocks (Table 1). Three blocks (1, 2, and 5 in Table 1) were pure practice blocks in which either target stimuli (Blocks 1 and 5) or attribute stimuli (Block 2) were sorted in their reference categories. The remaining blocks were the associative blocks that constitute the two mapping conditions of the IAT (e.g., mapping healthy-good and mapping sick-bad). Participants were trained how to differentiate between the sick and healthy images during Block 1. Each image was presented 3 times in random order, resulting in 24 trials. In Block 2, participants were instructed to use the same response keys to classify 8 good words and the 8 bad words presented 3 times, resulting in 48 trials. Block 3 was a combination of the two former asks. More concretely, during this block, half of the participants were asked to press the same key for healthy images and good words and the other half of the participants were asked to press the same key for healthy images and bad words. Each word was presented twice (2 × 16 = 32 trials), and each image was presented 4 times (4 × 4 = 32 trials), resulting in 64 trials. Words and images were presented randomly throughout the block. Block 4 was the same as Block 3 though the order of the trials was randomized. In Block 5, the target categories swapped their position on the screen. During this Block 5, the 4 healthy and the 4 sick images were presented three times (24 trials). This time, however, words from the good and bad categories were not presented. In Block 6, words and images were presented in a combined manner following the switched target location from Block 5. Each word was presented twice (2 × 16 = 32 trials), and each image was presented 4 times (4 × 4 = 32 trials), resulting in 64 trials. Block 7 was the same as Block 6 though the order of the trials was randomized.

**TABLE 1:** Block sequence in the Implicit Association Test (IAT).

**Block**	**# of trials**	**Function**	**Left-key response**	**Right-key response**
1	24	Practice	Healthy	Sick
2	48	Practice	Bad	Good
3	64	Associative	Healthy—Bad	Sick—Good
4	64	Associative	Healthy—Bad	Sick—Good
5	24	Practice	Sick	Healthy
6	64	Associative	Sick—Bad	Healthy—Good
7	64	Associative	Sick—Bad	Healthy—Good

**Blocks 3–4 and 6–7 associative order and key response assignment were counterbalanced between participants.**

During the task, the words/images were presented one by one in the center of the screen and participants had to indicate as quickly and accurately as possible, by pressing the corresponding response key on the keyboard, the category (healthy, sick, good, bad) to which each of them belonged. Category concepts were permanently visible in the left and right corners of the screen. Word categories were presented in blue and image categories in green. Participants were told to press the “e” (index finger left hand) for words/images from a category displayed in the left corner and the “i” (index finger right hand) for words/images from a category displayed in the right corner. A red cross appeared whenever the participant’s answer was wrong, which indicated the need to correct the mistake by quickly pressing the alternate key. When a correct response was provided the next word/image was displayed. Response latency (i.e., reaction time) was calculated from the moment a word/image appeared until the time the participant gave a correct response. Trials with a reaction time less than 400 ms were removed.

## Results

**FIGURE 1:**
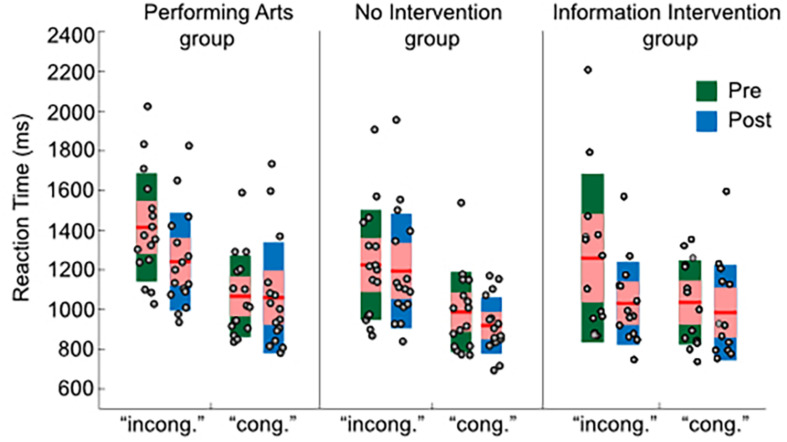
Latency results for each study group and condition. RT measures were extracted by averaging RTs to associative blocks in the IAT design. The RTs for each individual and individual and condition are represented in gray circles. For all boxplots, the central mark is the median, the edges of the box are the 25^th^ and 75^th^ percentiles.

To assess for the existence of changes in cognitive biases specifically in the experimental group between IAT sessions, a mixed ANOVA was performed on mean reaction time (RT) to the associative blocks (Figure 1). The ANOVA included congruency (2 levels: congruent vs. incongruent) and session (2 levels: pre and post) as within subject factors and group (3 levels: experimental group, no-intervention control group and information intervention control group) as between subject factor. This analysis revealed a statistically significant main effect of session [*F*(1, 43) = 12.43, *p* = 0.001, η^2^ = 0.22] and congruency [*F*(1, 30) = 28.43, *p* < 0.001, η^2^ = 0.39], corroborating that participants were slower in categorizing words/images when social biased categories shared the location on the screen (i.e., sick/good and healthy/bad). Importantly, we found a triple congruency, session and group trend toward significant effect [*F*(2, 43) = 2.73, *p* = 0.07, η^2^ = 0.11] but not a session × group [*F*(2, 43) = 0.96, *p* = 0.39, η^2^ = 0.04] nor congruency × group interaction effect [*F*(2, 43) = 0.98, *p* = 0.38, η^2^ = 0.04], suggesting that the congruency effects between conditions and sessions varied differently as a function of the study group. To identify the source of this effect, we ran a repeated measures ANOVA including congruency and session as within subject factors separately for each group. This analysis showed a significant main effect of congruency in the experimental group [*F*(1, 15) = 16.92, *p* = 0.001, η^2^ = 0.53], in the no-intervention control group [*F*(1, 15) = 16.26, *p* = 0.001, η^2^ = 0.52] but not in the information control group [*F*(1, 13) = 2.46, *p* = 0.14, η^2^ = 0.16]. We also found a main effect of session in the experimental group [*F*(1, 15) = 5.54, *p* = 0.03, η^2^ = 0.27] and a trend toward statistical significance in the no-intervention control group [*F*(1, 15) = 3.39, *p* = 0.08, η^2^ = 0.18] and in the information intervention control group [*F*(1, 13) = 4.19, *p* = 0.06, η^2^ = 0.24]. Importantly, however, this analysis revealed a significant session x congruency interaction effect in the experimental group [*F*(1, 15) = 10.32, *p* = 0.006, η^2^ = 0.41] but not in the no-intervention control group [*F*(1, 15) = 0.31, *p* = 0.58, η^2^ = 0.02] nor in the information intervention control group [*F*(1, 13) = 0.30, *p* = 0.11, η^2^ = 0.19]. A student paired *t*-test analysis confirmed that the session x congruency interaction in the experimental group was driven by differences between sessions for the incongruent [*t*(15) = 3.01, *p* = 0.009, Cohen’s *d* = 0.75] rather than for the congruent [*t*(15) = 0.23, *p* = 0.82; Cohen’s *d* = 0.06] trials. To further assess for the specificity of the congruency effects found in the critical experimental condition, we ran a mixed ANOVA with the outcome variable mean reaction time (RT) to the practice blocks. The ANOVA included Block (2 levels: 1st vs. 2nd presentation block) and session (2 levels: pre and post) as within subject factors and group (3 levels: experimental group, no-intervention control group and information intervention control group) as between subject factors. This analysis revealed a significant main effect of Block [*F*(1, 43) = 9.80, *p* < 0.01, η^2^ = 0.19] and session [*F*(1, 43) = 27.79, *p* < 0.01, η^2^ = 0.39], but no interaction with group (all ps > 0.2, all η^2^ < 0.1).

Finally, we calculated the IAT *D*-score, which instead of comparing within-participant differences in RTs, standardizes them at the participant level, dividing the within-participant difference by a “pooled” standard deviation (Greenwald et al., 2003). *D*-scores in the experimental group were *M* = 0.73 (*SD* = 0.86) and *M* = 0.46 (*SD* = 0.71) in the pre and post observation, respectively, *M* = 0.41 (*SD* = 0.56) and *M* = 0.55 (*SD* = 0.44) in the pre and post measures in the no-intervention control group and *M* = 0.36 (*SD* = 0.67) and *M* = 0.14 (*SD* = 0.69) in the pre and post measures in the information intervention control group. A mixed ANOVA, including session (2 levels: pre and post) as a within-subject factor and group (3 levels: experimental group, no-information control group and information intervention control group) as a between-subject factor, confirmed previous findings by revealing a trend toward statistically significant session x group interaction [*F*(2, 43) = 2.74, *p* = 0.08, η^2^ = 0.12]. A paired student *t*-test showed statistically significant *D*-score difference between pre and post in the experimental group [*t*(15) = 2.55, *p* = 0.02, Cohen’s *d* = 0.64] but not in the no-information control group [*t*(15) = −1.04, *p* = 0.32, Cohen’s *d* = −0.26] nor in the information intervention control group [*t*(13) = 1.27, *p* = 0.23, Cohen’s *d* = 0.34].

## Discussion

The aim of the current study was to assess whether the involvement in a performing arts training program of 14 full days could lead to a shift in implicit measures. To this end, we conducted an experiment in which healthy participants completed an IAT to assess biased attitude to illness in two separate sessions, before and after the training program. A similar two-session IAT task was completed by two different matched control groups of healthy participants. Results showed that participants who were involved in the training program reduced their implicit bias toward illness measured through IAT in the second session. This reduction in IAT measures was not observed in the control samples, despite the two IAT measures being matched in temporal delay with the experimental group. These findings suggest that an interventional program based on the performing arts could be effective in reducing levels of implicit biases among the general population.

To the best of our knowledge, this is the first study showing that a training program based on acting can have an influence on implicit cognitive bias, thereby raising the possibility of extending previously established lab-based interventional techniques that can reduce implicit bias (e.g., Lai et al., 2014, 2016) to include more realistic approaches. Current findings therefore may represent a first attempt to show the promising possibilities that the performing arts may have to help treat prejudice and stereotypes. At the same time, the present study lacks a clear explanation of the mechanisms that underlie this effectiveness, as it was not designed with such purpose. Nevertheless, we can offer some speculations. Previous research revealed that most effective lab-based techniques to reduce implicit bias invoke self-relevancy, emotion and vividness (Lai et al., 2014). While designing strategies that can effectively elicit these properties in individuals may be challenging in the laboratory, inducing them by requesting people to perform as actors seems more practicable. Thus, one plausible explanation of the changes in IAT in our study was driven by the self-involvement of the participants during the training program, which in turn, induced a more thorough processing of the scenario. An alternative explanation would be that participants that engaged the performing arts training program increased their general cognitive control skills, which has been shown to influence implicit bias tasks (e.g., Payne, 2001, 2005; Amodio et al., 2008; Klauer et al., 2010). Future studies may include separate measures of executive functioning (e.g., Stroop task) in conjunction with the implicit bias tasks to be able to assess bias- and control-related ability changes due to an interventional program.

Several important questions, however, remained unanswered in the current study. The first one concerns the specificity of the effects. More concretely, it would be important to determine in the future the extent to which the reduction in implicit bias was specific to the stereotyped concept that surrounds the training program. For example, one would expect the reduction in implicit bias to be specific to illness but not to other stereotypes such as racial or gender bias. Our findings showing that only the experimental group, but not the control groups, had a reduction in implicit measures speaks about the effectiveness of the interventional program but not about its specificity at the implicit bias level. Future research may include different IAT versions at the within-subject design to scrutinize this possibility.

A second concerns refers to the small sample size of the experimental groups in our study. Though we believe current findings are well supported by effect sizes, the within-subject design and the inclusion of two control groups, future studies aiming to assess the impact of the performing arts in implicit biases should, if possible, include larger sample sizes. This would increase the statistical power of the study, thereby ensuring the effects can be extrapolated to the overall population, especially if the expected effects cannot be assessed at the within-subject level.

Another important issue is to assess the extent to which the changes in implicit bias observed in the current design persist over time. Previous research revealed that stability of changes in implicit preferences are difficult to observe in available interventional techniques (Lai et al., 2016; see, however, Peck et al., 2013). While the current research does not look at long-term effectiveness, our proposed framework engaged participants in a 14-days training program unlike previous interventional programs that involved a single session at the lab. At the speculative level, we hypothesized that engaging the participants in a consecutive day-to-day program may promote memory consolidation of learned non-biased association between concepts. A consequence of such a consolidation process would be participants’ reduction in implicit bias being accompanied by an explicit awareness of the bias. Indeed, previous research on memory consolidation showed that night sleep promotes knowledge awareness (e.g., Wagner et al., 2004). If that were the case, it would suggest that an interventional program has an impact in transforming implicit to explicit memory representations. A possible advantage of such transformation is that it may thus favor generalization (Ranganath and Nosek, 2008), thereby promoting implicit bias reduction in other conceptual domains. Future studies should examine this possibility by combining IAT measures with questionnaires that help assess attitude awareness of cognitive bias.

Society is interested in reducing the implicit biases in the general population. So far, most of the interventional techniques described in the literature that may help reduce implicit bias have been developed to be implemented in the lab. Though lab-based techniques offer experimental rigor and control, they may limit participants’ motivation and engagement with a task, thereby complicating the possibility of making reliable changes in implicit bias at an individual level. An alternative strategy is to take advantage of realistic activities that invoke solid individual self-engagement with a task, with the hope that this fosters strong changes at an implicit level. Current research underscores the powerful impact that performing theater can have in reducing implicit bias. Our findings show that implicit bias toward physical illness is reduced in a group of non-professional artists after enrolling in a 14-days training program based on theater acting. The extent to which such an interventional program has a causal effect and if so, whether such effect is specific to a specific conceptual bias (i.e., physical illness) remains to be examined in the future.

## Data Availability Statement

The datasets generated for this study are available on request to the corresponding author.

## Ethics Statement

The studies involving human participants were reviewed and approved by the University of Barcelona’s Bioethics Commission. The patients/participants provided their written informed consent to participate in this study.

## Author Contributions

JG-A, PC, and LF contributed to the conception and design of the study. JG-A and PA programmed the computer task. PA and CV-G acquired the data. JG-A and LF performed the statistical analysis and wrote the first draft of the manuscript. All authors contributed to manuscript revision, read, and approved the submitted version.

## Conflict of Interest

The authors declare that the research was conducted in the absence of any commercial or financial relationships that could be construed as a potential conflict of interest.

## Footnotes

1 https://epicalab.com

2 https://www.youtube.com/watch?v=LtalG87Epsg
